# Locked and Loaded: β-Galactosidase Activated Photodynamic Therapy Agent Enables Selective Imaging and Targeted Treatment of Glioblastoma Multiforme Cancer Cells

**DOI:** 10.1021/acsabm.2c00484

**Published:** 2022-08-31

**Authors:** Toghrul Almammadov, Zubeyir Elmazoglu, Gizem Atakan, Dilay Kepil, Guzide Aykent, Safacan Kolemen, Gorkem Gunbas

**Affiliations:** † Department of Chemistry, Koç University, Rumelifeneri Yolu, 34450 Istanbul, Turkey; ‡ Department of Chemistry, Middle East Technical University (METU), 06800 Ankara, Turkey; § Surface Science and Technology Center (KUYTAM), Koç University, 34450 Istanbul, Turkey; ∥ Boron and Advanced Materials Application and Research Center, Koç University, 34450 Istanbul, Turkey

**Keywords:** Targeted PDT, glioblastoma, theranostics, resorufin, cancer cell selectivity, β-galactosidase

## Abstract

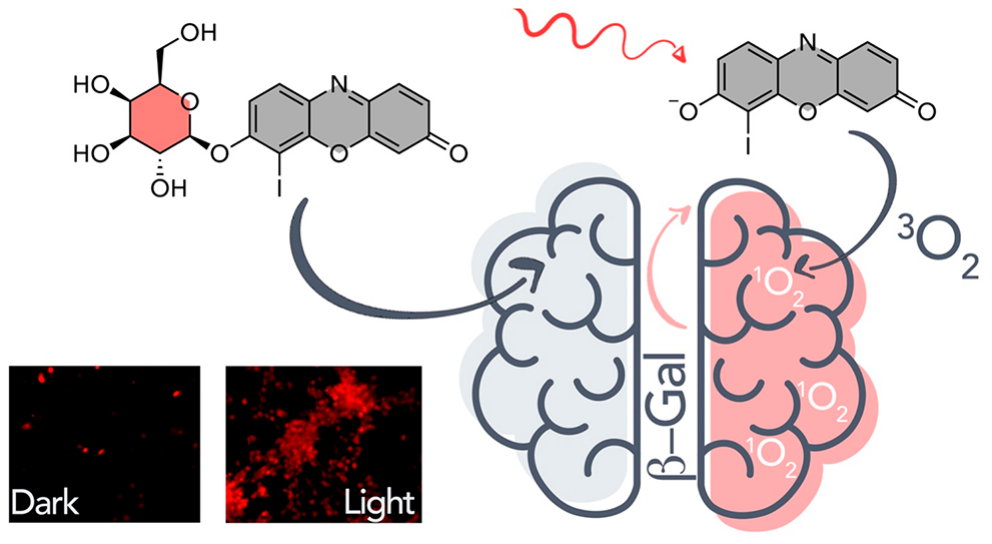

Selective detection and effective therapy of brain cancer, specifically, the very aggressive glioblastoma multiforme (GBM), remains one of the paramount challenges in clinical settings. While radiotherapy combined surgery is proposed as the main treatment course, it has several drawbacks such as complexity of the operation and common development of recurrent tumors in this course of patient care. Unique opportunities presented by photodynamic therapy (PDT) offer promising, effective, and precise therapy against GBM cells along with simultaneous imaging opportunities. However, activatable, theranostic molecular systems in PDT modality for GBM remained scarce. Specifically, even though elevated β-galactosidase (β-gal) activity in glioblastoma cells is well-documented, targeted, activatable therapeutic PDT agents have not been realized. Herein, we report a β-galactosidase (β-gal) activatable phototheranostic agent based on an iodinated resorufin core (**RB-1**) which was realized in only three steps with commercial reagents in 29% overall yield. **RB-1** showed very high singlet oxygen (^1^O_2_) quantum yield (54%) accompanied by a remarkable turn-on response in fluorescence upon enzymatic activation. **RB-1** was tested in different cell lines and revealed selective photocytotoxicity in U-87MG glioblastoma cells. Additionally, thanks to almost 7% fluorescence quantum yield (Φ_F_) despite extremely high ^1^O_2_ generation yield, **RB-1** was also demonstrated as a successful agent for fluorescence imaging of U-87MG cells. Due to significantly lower (β-gal) activity in healthy cells (NIH/3T3), **RB-1** stayed in a passive state and showed minimal photo and dark toxicity. **RB-1** marks the first example of a β-gal activatable phototheranostic agent toward effective treatment of glioblastoma.

## Introduction

Photodynamic therapy (PDT) is a highly promising treatment approach, which has gained increasing interest during past two decades as an alternative to state-of-the-art therapies.^1−3^ In a typical PDT process, therapeutic action is realized by the generation of highly cytotoxic and short-lived reactive oxygen species (ROS) and singlet oxygen (^1^O_2_), after excitation of photosensitizers (PSs) by a proper light source, operating preferably in the red or near-IR region. In addition to oxidative damage on vital biomolecules caused by the high reactivity of ^1^O_2_ and/or ROS, PDT also activates the immune system against cancer cells.^4,5^ Furthermore, intrinsic selectivity that is arising from the delivery of light to the tumor region and opportunities for repeated applications with nondeveloping drug resistance are all strongly contributing factors for the success of the therapeutic outcome.^6,7^ New generation PSs are also capable of inducing photocytotoxicity solely in cancer cells, without harming the normal tissues even under light irradiation through targeting and/or activation strategies.^8−11^ Thus, they further enhance the inherent selectivity of PDT action and eliminate the potential side effects caused by unwanted phototriggered reactions. In this direction, activity-based PSs (aPSs) have appeared to be an attractive candidate toward the development of cancer cell selective PDT agents.^12−15^ aPSs can trigger ROS generation after they are activated by tumor associated stimuli including but not restricted to overexpressed enzymes, a high level of small biomolecules (e.g., biothiols, reactive oxygen/nitrogen species), and the acidic microenvironment of cancer cells.^16−20^ On the contrary, they tend to stay in their OFF state in normal cells.

In the design of aPSs, several ROS quenching strategies can be utilized.^21−23^ In all of these approaches, the choice of the PS core is highly critical. It should not only allow design flexibilities and ease of modification but also needs to hold unique characteristics such as strong absorption coefficients, water solubility, high ^1^O_2_ generation yield in aqueous solutions, photostability, and low dark toxicity. Recently, we reported that iodinated resorufin derivatives, which fulfill all of the mentioned requirements, can serve as a highly effective PS.^24^ We showed that masking the phenol unit of the resorufin core with a cleavable cage unit allows modulation of excited state dynamics through an ICT process enabling the design of aPSs, which can be selectively activated with a wide range of analytes.^24,25^

Glioblastoma (GBM) is a highly aggressive and lethal type of glioma, which has a very poor prognosis and high incidence rate.^26^ The current state-of-the-art treatment method for GBMs involves surgery for complete resection of the tumor, which by itself is a daunting task, since GBM cells are highly proliferative and can easily migrate away from the tumor center.^27^ The surgery, in most cases, is followed by combined application of fractionated radiation therapy and chemotherapy.^28^ There are several chemotherapy drugs utilized for GBM treatment in the clinic including Temozolomide, Procarbazine, Carmustine, and Lomustine, however none of those were proven to be successful in avoiding the recurrence of the tumor around the resection area and only minimally (in range of months) extend life expectancy.^29^ Additionally, severe side effects of this modality of treatment are well documented.

Recent advances in the field of PDT such as interstitial PDT and emerging new generation PSs along with the unique advantages of PDT compared to conventional therapies have rejuvenated the interest toward its usage against brain tumors, specifically to inhibit recurrence in the resection cavity. To date, the most popular PS that has been applied in this direction in clinical trials is 5-ALA, which holds FDA approval for fluorescence guided surgery applications.^30,31^ Although, 5-ALA offers selective uptake in gliomas,^31^ most of the first-generation PSs do not offer targeted therapy opportunities and suffer significantly from their highly hydrophobic nature. Thus, there is still a need for new PS designs that can selectively induce photocytotoxicity in brain cancer cells, while eliminating off-target photosensitization in healthy cells. Although selective fluorescent imaging of GBM and other gliomas has advanced significantly in recent years via utilization of molecular probes, brain-cancer-cell-targeted PDT agents have remained elusive. In a few recent examples, two different methylene blue (MB)-based PSs that can be activated by γ-glutamyl transpeptidase (GGT) were reported to selectively ablate glioma and GBM cells that are known to exhibit high GGT activity.^32^ Unfortunately, MB-based PSs mostly suffer from high dark toxicity, and the MB core can be reduced to inactive leuco-methylene blue in a cellular environment.^33^ β-galactosidase (β-gal) is a popular enzyme, which plays critical roles in energy production as it catalyzes the cleavage of disaccharide lactose to monosaccharides.^34^ On the contrary, abnormal levels of β-gal are directly associated with different cancer types, specifically metastatic ovarian cancer.^35^ It was also reported that β-gal activity is significantly enhanced in gliomas including GBM, especially in primary tumors, compared to normal cells, which is also associated with malignancy.^36^ Given that β-gal is a distinct tumor biomarker, it has attracted great attention in bioimaging studies, and much effort has been put forward for visualization of fluctuations of the β-gal level with fluorescent probes for diagnostic purposes.^37−42^ A similar approach was also utilized for designing prodrugs to get selective chemotherapy agents.^43,44^ However, β-gal activatable phototherapeutic agents remained rare, surprisingly,^45−48^ and there is no report on a β-gal responsive PDT agent designed for selective treatment of brain cancers.

**Figure 1:**
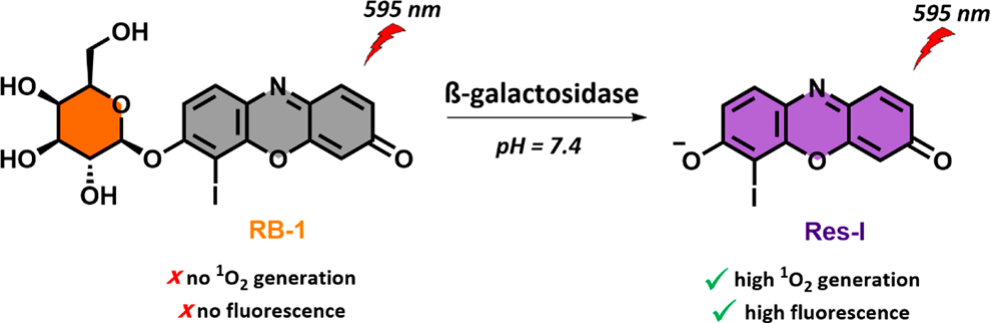
Structure of **RB-1** and its activation upon addition of β-gal.

Resorufin, a long known fluorescent reporter,^49^ has been recently converted to a PDT agent by incorporating iodine atoms to enhance heavy atom-mediated spin orbit coupling (SOC), which consequently triggers effective intersystem crossing (ISC).^24,25^ A heavy atom decorated resorufin core as a PS was shown to demonstrate highly sought characteristics including low toxicity in the dark, a high extinction coefficient in the red region, photostability, high ^1^O_2_ quantum yield, and amphiphilicity, which make it an attractive PDT agent. It was shown that iodo-resorufin has 61% singlet oxygen quantum yield in aqueous solutions, which is comparable to the standard PSs that are frequently used in the literature.^24^ Additionally, iodo-resorufin maintains its fluorescence emission, even though the ISC path is predominant after heavy atom addition and acts as a phototheranostic agent.^24^ Importantly, the 7-hydroxy group of the PS core can be easily masked with a cleavable cage unit toward the development of an aPS. Herein, we modified the iodo-resorufin core with a β-gal responsive sugar unit to design a PDT agent (**RB-1**) that can be selectively activated in glioblastoma cells (Figure 1). **RB-1** tends to stay in its OFF state prior to β-gal activation and does not show any photocytotoxicity and fluorescence signal. A high β-gal level in GBM cells initiates the hydrolysis of glycosidic linkage between β-d-galactopyranose masking unit and the PS core, which releases the highly cytotoxic and emissive iodo-resorufin core (Res-I; Figure 1). Thus, it was shown here, for the first time, that β-gal overexpression in GBM cells can be utilized for therapeutic purposes.

## Result and Discussion

### Synthesis of **RB-1**

**Figure 2:**
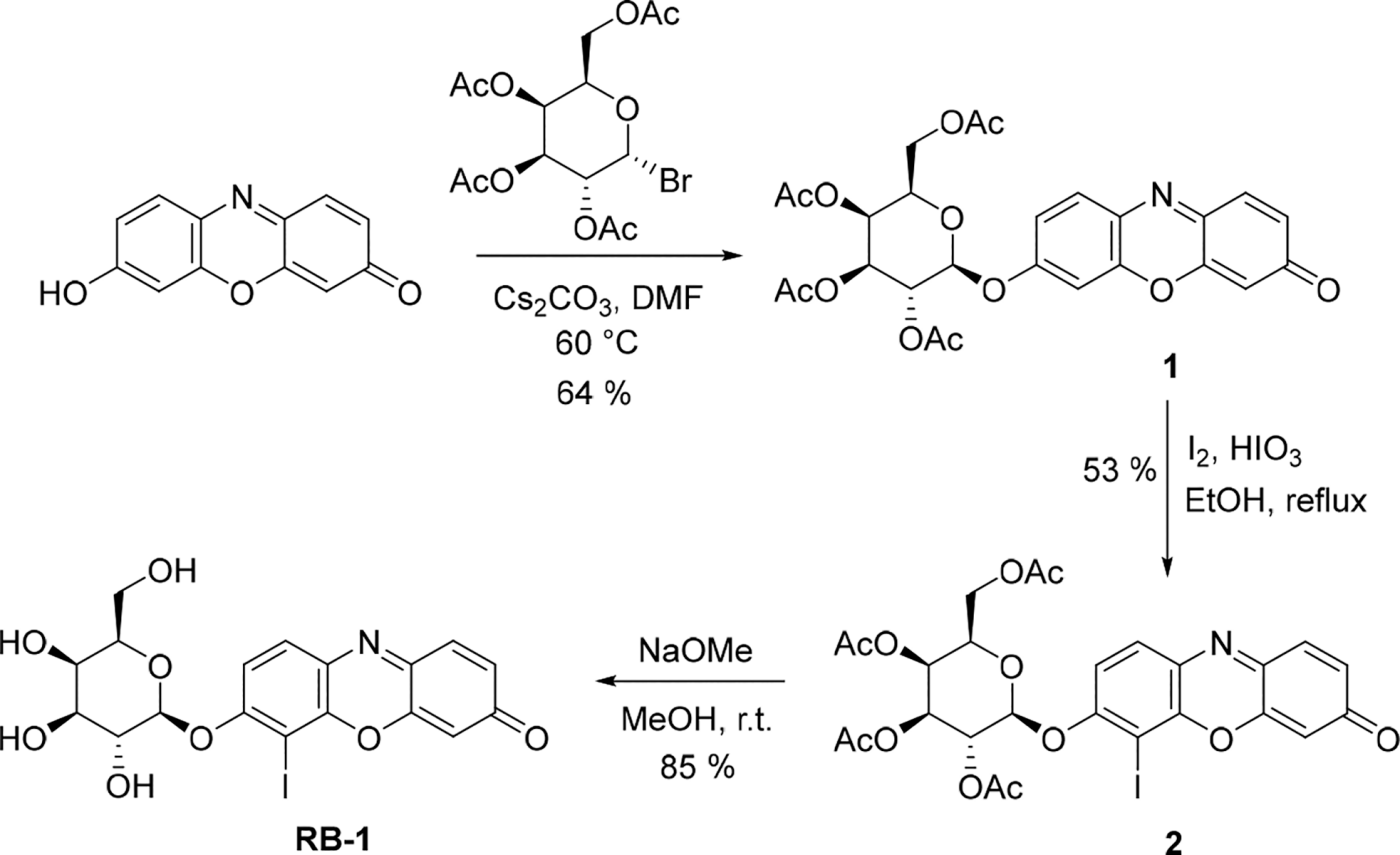
Synthetic pathway of **RB-1**.

The synthesis of **RB-1** started with the reaction between commercially available resorufin and 2,3,4,6-tetra-*O*-acetyl β-galactopyranosyl bromide in the presence of Cs_2_CO_3_ to produce **1**. Then, iodine was inserted into the core structure by the addition of I_2_ and HIO_3_ in ethanol, which yielded compound **2**. In the last step, deacetylation of the hydroxy groups by NaOMe gave water-soluble resorufin-based **RB-1** with an overall yield of 29% (Figure 2). A high purity (97%) of **RB-1** was demonstrated by RP-HPLC (Figure S1).

### Optical Characterizations

**Figure 3:**
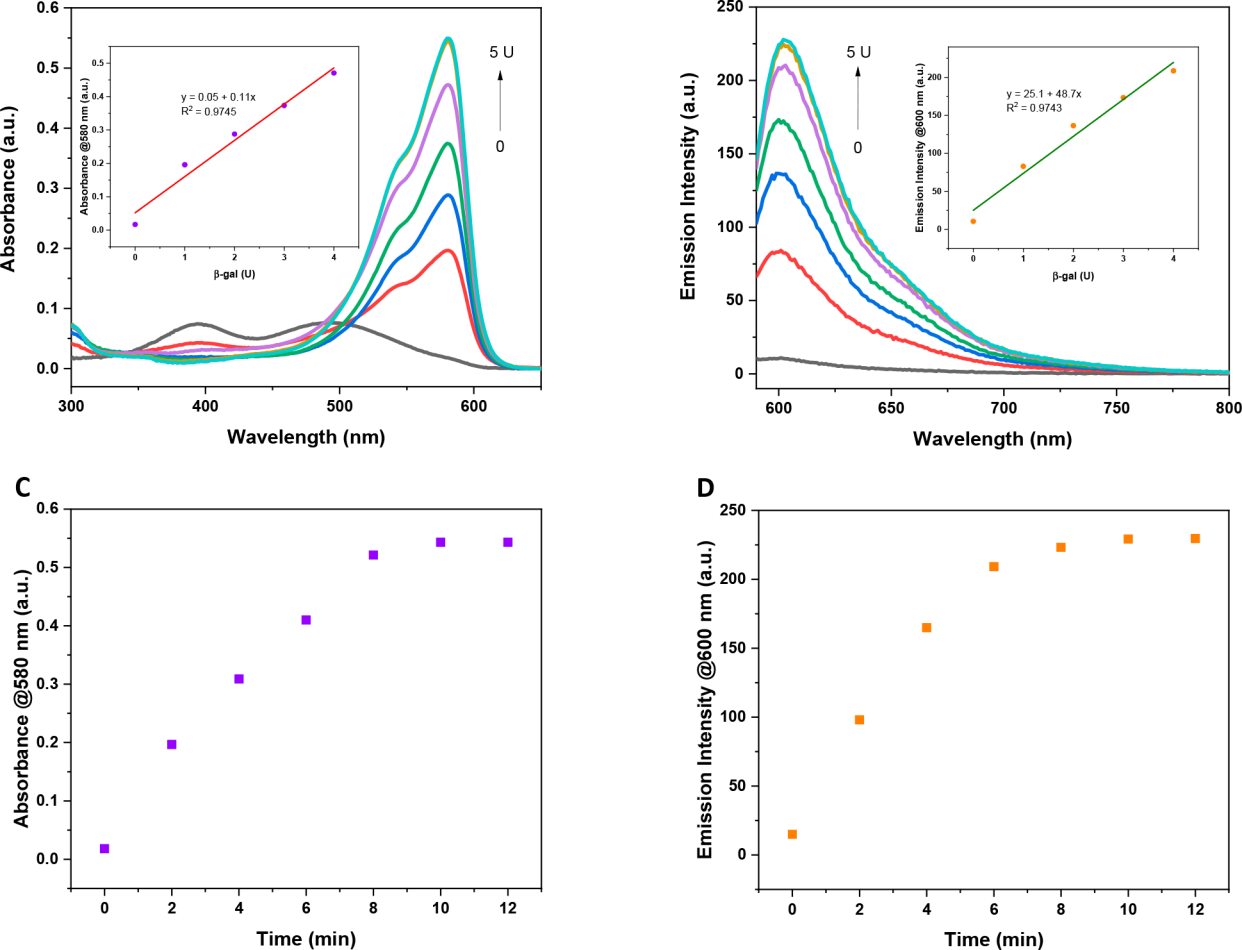
(a) UV–vis and (b) emission spectra of **RB-1** (10 μM) upon the addition of β-gal (0–5 U). Insets: UV–vis and fluorescence signals at 580 and 600 nm, respectively, versus β-gal concentrations in the linear range. In each case, **RB-1** (10 μM) and β-gal were mixed for 10 min. Time dependent change in the (c) UV–vis and (d) emission signals of **RB-1** (10 μM) after adding 5U β-gal. λ_ex_ = 580 nm.

We first determined the response of **RB-1** to β-gal by acquiring absorption and fluorescence spectra in PBS (1% DMSO, pH 7.4). **RB-1** exhibited two signals, which were centered at 490 and 400 nm, with a negligible fluorescence signal (Figure 3a). After the addition of increased β-gal concentrations (0–5 U), a single and sharp red-shifted absorption signal centered at 580 nm that belongs to the parent iodo-resorufin (Res-I) core (Figures 3a, S3) emerged gradually.^24^ Similarly, a concentration dependent increase (22-fold) in the emission signal was detected at 600 nm (Figure 3b, S3), which is consistent with the parent Res-I emission signal. Φ_F_ was calculated as 6.8% for β-gal treated **RB-1** by employing sodium salt resorufin as a reference dye, which is quite low compared to the regular resorufin core due to effective ISC (Table 1). Additionally, it was shown that cage cleavage can be completed in 10 min after the addition of the enzyme (Figure 3c,d). To further prove that Res-I was released as a result of enzymatic reaction, HPLC analyses were also performed. Upon treating **RB-1** with different concentrations of β-gal, a new product was observed that eluted at the same time (*t* = 7.56 min) with the Res-I core, clearly suggesting enzymatic cleavage of the masking unit and the release of active Res-I (Figure S2). **RB-1** was further treated with different analytes to test its selectivity. No significant change was detected in absorption spectra of **RB-1** in the presence of all analytes tested, indicating high selectivity toward β-gal (Figure S4).

**Table 1:** Photophysical Characteristics and ^1^O_2_ Generation Yields of the β-gal Treated and Untreated **RB-1**

	λ_abs_ [nm]^a^	ε [M^–1^ cm^–1^]^a^	λ_ems_ [nm]^a^	Φ_F_ [%]^a^^,^^b^	Φ_Δ_ [%]^a^^,^^c^
**RB-1**	490, 400	9000, 7600	600	n.d.^d^	n.d.^d^
**RB-1** + β-gal	580	54000	600	6.8	54

a In PBS (pH 7.4, 1% DMSO).

b Reference: resorufin sodium salt in PBS (Φ_F_ = 0.74).^49^

c Reference: methylene blue in PBS (Φ_Δ_ = 0.52).^50^

d Not determined.

**Figure 4:**
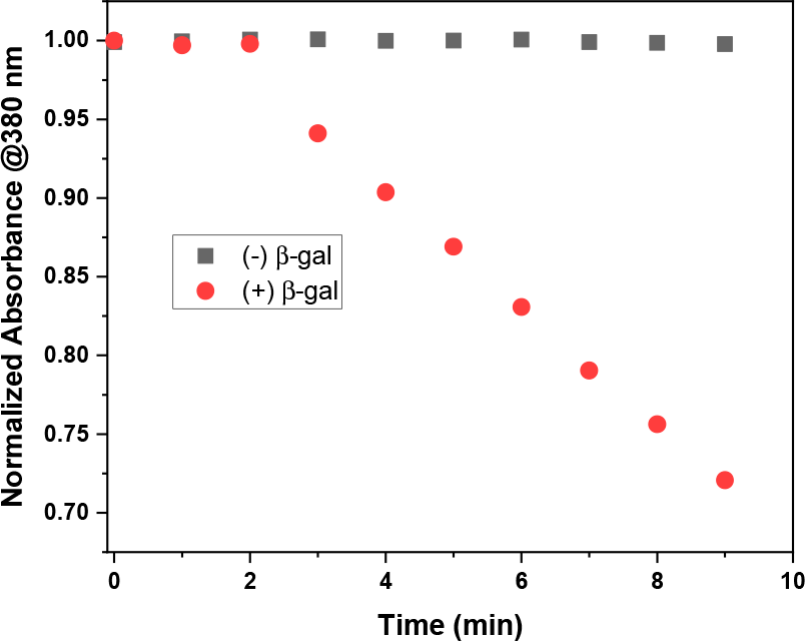
Relative ^1^O_2_ generation of **RB-1** before and after the addition of 5U β-gal in PBS buffer (pH = 7.4, 1% DMSO). Samples were kept in the dark for the first 2 min.

Next, the ^1^O_2_ generation capacity of **RB-1** was investigated by utilizing commonly employed ^1^O_2_ trap molecule 2,2′-(anthracene-9,10-diyl)bis(methylene)dimalonic acid (ADMDA). Upon irradiation of β-gal-treated **RB-1** with 595 nm LED (9.83 mW/cm^2^) light, a time-dependent decrease was observed in the absorption peak of the trap molecule at 380 nm. This suggests the cycloaddition reaction between photosensitized ^1^O_2_ and the anthracene ring (Figure 4). On the contrary, no change was observed in the absorption signal, when **RB-1** was irradiated without enzyme addition as the PS cannot absorb the excitation light (Figure 4). Singlet oxygen yield for β-gal-treated **RB-1** was calculated as 54% (reference: methylene blue Φ_Δ_ = 0.52 in PBS buffer).^50^

### Cytotoxicity

**Figure 5:**
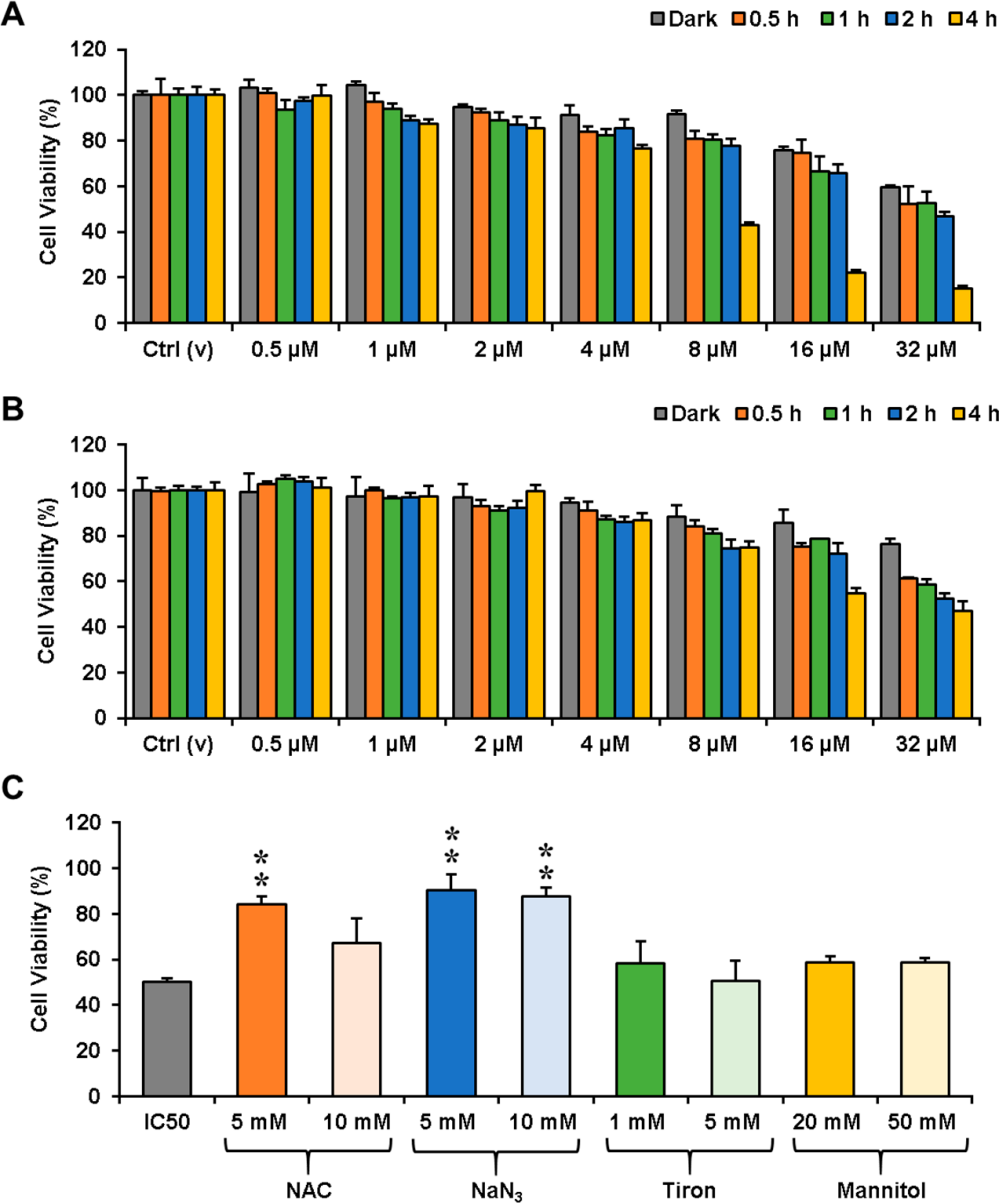
Changes in the cell viabilities of U-87MG (A) and NIH/3T3 (B) treated with varying concentrations of **RB-1** (0.5–32 μM). Cells either kept in the dark for 24 h or for 0.5–4 h in the dark, then irradiated for 2 h (*n* = 6). (C) The cell viabilities of U-87 MG treated with the IC_50_ values (7.7 μM) of **RB-1** for 4 h in the dark, then irradiated for 2 h in the presence of NAC, NaN_3_, Tiron, or Mannitol (*n* = 5–6). Ctrl (v), vehicle control; NAC, N-acetlycsyteine; NaN_3_, sodium azide. *p < 0.05, **p < 0.01 vs IC_50_.

The potency of **RB-1** as a phototherapeutic agent was investigated in cancerous U-87MG (GBM) and normal NIH/3T3 cells by utilizing a conventional MTT (methyl thiazolyl tetrazolium) assay. Both cells were incubated with **RB-1** for 30 min, 1 h, 2 h, or 4 h at varying concentrations (0–32 μM) and then irradiated with an LED (595 nm, 9.83 mW/cm^2^). A control group was kept in the dark for 24 h prior to MTT assay, and a noticeable decrease in cell viability was only observed in U-87MG cells at the highest dose of **RB-1** (32 μM), suggesting low dark toxicity in a wide range of concentrations (Figure 5a,b). **RB-1** did not show effective photocytotoxicity up to a 16 μM dose under light irradiation after 0–2 h of incubation periods in both cell lines (Figure 5a,b). Incubation of the agent for an additional 2 h and subsequent light irradiation for 4 h triggered a remarkable drop in cell viability in U-87MG cells (IC_50_ = 7.7 μM; Figure 5a), which can be attributed to better uptake and a more efficient activation process. It is important to note here that quite a low power LED light (9.83 mW/cm^2^) array has been used for these studies, which is the main reason for the relatively longer irradiation times. A much faster response can always be achieved with higher power laser sources. The survival rate for NIH/3T3 remained at a very high level even at a 32 μM **RB-1** dose, suggesting that the agent can induce noteworthy differential photocytotoxicity between cancerous and healthy cells (Figure 5b). Later, several inhibition studies were performed in U-87MG cells, while keeping **RB-1** at the IC_50_ dose to evaluate the type of ROS generated during the PDT action (Figure 5c). Treating cells with NaN_3_, a well-known singlet oxygen quencher,^51^ dramatically increased the cell viability, suggesting that ^1^O_2_ is the primary cytotoxic agent. Similar results were obtained in *N*-acetyl cysteine (NAC) treated cells, as NAC is a general ROS scavenger.^52^ On the other hand, mannitol and tiron quenches the hydroxyl radical^53^ and superoxide anion,^54^ respectively, and addition of these inhibitors made a slight impact on the survival rate, once again proving that therapeutic action depends on effective ^1^O_2_ generation (Figure 5c).

### Intracellular ROS Detection and Cell Death Mechanism

**Figure 6:**
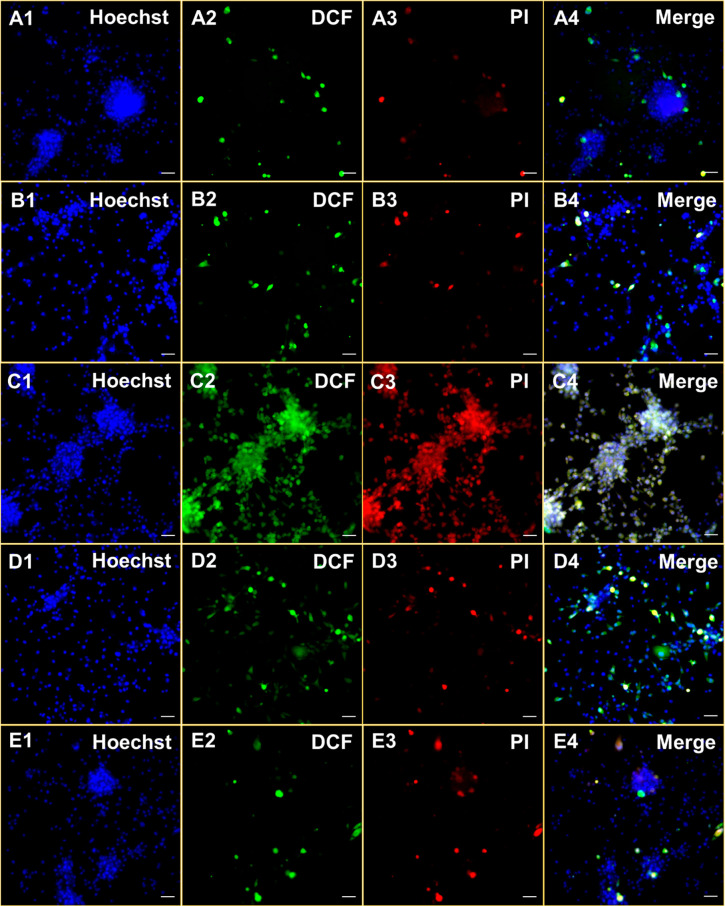
Hoechst/DCF-DA/PI triple staining of U-87MG cells either incubated with DMSO (0.2%; A1–A4) or **RB-1** (7.7 μM) under dark conditions (B1–4), under LED irradiation in the absence (C1–C4) or presence of NAC (10 mM; D1–D4) or NaN_3_ (10 mM; E1–E4) for 2 h. Scale bar: 50 μm. Blue, Hoechst 33342; green, DCF; red, PI. DCF, 2′-7′-dichlorofluorescein; PI, propidium iodide.

The ROS sensor 2′,7′-dichloro-fluorescein diacetate (DCFH_2_-DA) was used to verify the intracellular ROS formation. DCFH_2_-DA bears two acetate groups, which are cleavable by intracellular esterases, making it cell trappable. The resulting molecule is oxidized to dichlorofluoroscein (DCF) upon reacting with ROS, which displays characteristic green emission upon light irradiation.^55^ Additionally, cells were treated with a red emitting propidium iodide (PI) to visualize death cells.^56^ When **RB-1**-incubated U-87MG cells were subjected to light irradiation, both DCF and PI emission were detected, revealing effective ROS production and consequent cell death (Figure 6). In the case of NAC and NaN_3_ treated cells, green DCF emission was quenched, suggesting again that ^1^O_2_ is the primary source of ROS (Figure 6), which is in good correlation with the cell viability results. Suppressed ROS generation in inhibition experiments increased the ratio of alive cells, which diminishes the PI emission. A low intensity signal in both channels was obtained from the control cells, which were either missing the agent or kept under dark conditions (Figure 6).

Next, we costained U-87MG cells with acridine orange (AO) and ethidium bromide (EtBr) to analyze the cell death mechanism in detail. Green emitting AO stains viable cells, whereas red emitting EtBr gives a red emission if the cells are dead. **RB-1** incubated cells displayed a reddish orange emission signal in the merge channel upon light irradiation, indicating that a majority of the cells underwent late apoptosis/necrosis (Figure S5). As expected, when cells were treated with either NAC or NaN_3_, only AO was activated, as most of the cells were alive. Similarly, green emission was observed in the control groups (Figure S5).

### Cell Imaging

**Figure 7:**
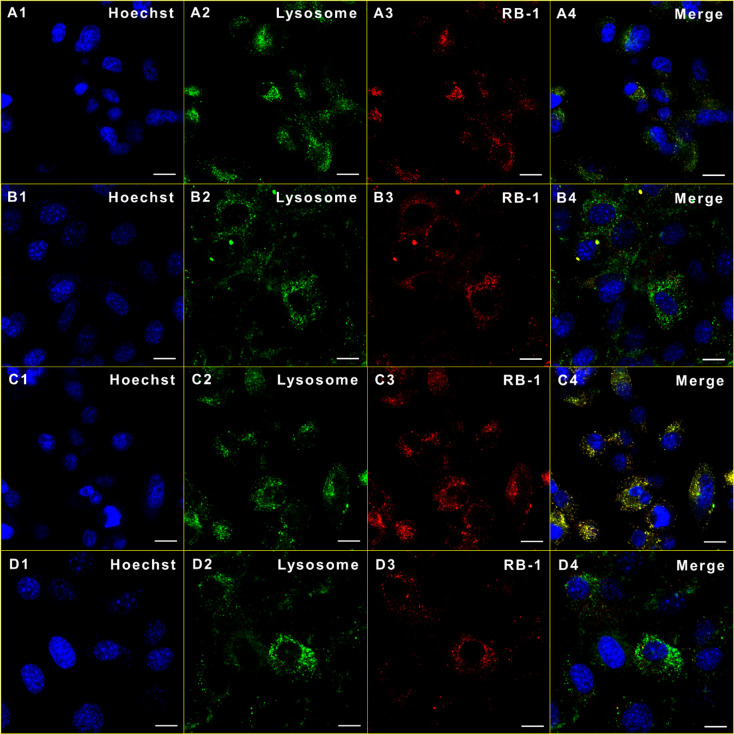
Cellular localization of **RB-1** (2 μM) in U-87MG (A, C) and NIH/3T3 (B, D) upon 30 min (A1–A3, B1–B3) or 4 h (C1–C3, D1–D3) incubation. Blue, Hoechst 33342; green, Lysotracker; red, **RB-1**. Scale bar: 10 μm.

Given that **RB-1** is highly emissive after getting activated by β-gal, its imaging capacity was evaluated under confocal microscopy. U-87MG and NIH/3T3 cells were incubated with **RB-1** (2 μM) either for 30 min or for 4 h. Cells were then washed and treated with Hoechst 33342 for nuclei staining. Additionally, to observe the intracellular localization of **RB-1**, U-87MG cells were also stained with a Lysotracker for lysosome imaging. Lysosome was specifically chosen as it was well established that β-gal is a typical lysosomal glycosidase.^36^ Thus, it is very likely for **RB-1** to get activated predominately in lysosome. As expected, **RB-1** showed a clear lysosomal emission in both cell lines with a stronger signal in cancerous U-87MG cells in accordance with the cell viability experiments (Figure 7). Furthermore, it was shown that a longer incubation period (4 h) resulted in stronger fluorescence compared to a 30 min treatment (Figure 7). We further investigated the time and dose dependent internalization of **RB-1** by following the fluorescence intensity of active Res-I. To this end, varying concentrations (0.5–8 μM) of **RB-1** were incubated with U-87MG cells, and confocal images were taken at different times. A dose dependent fluorescence intensity increase was observed, which appeared to be more pronounced after 4 h of incubation (Figure S6). This supports the better performance of the agent after a longer incubation period. These cumulative results suggest that **RB-1** can serve as an activatable phototheranostic agent in β-gal overexpressing cancer cells.

## Conclusion

To sum up, here we developed the first β-gal responsive phototheranostic agent (**RB-1**) for selective treatment and imaging of glioblastoma cells. **RB-1** was obtained after three high yielding steps and displayed a remarkable turn-on response in its fluorescence signal along with a remarkable ^1^O_2_ quantum yield in an aqueous solution upon reacting with the β-gal enzyme. **RB-1** was tested in cell culture studies, and selective photocytotoxicity was detected in U87 cancer cells with a negligible dark toxicity even at high doses. **RB-1** was also used to monitor lysosomal β-gal activity thanks to its relatively strong emissive character. Our results demonstrated that a high β-gal level in gliomas can be utilized as a promoter in the design of therapeutics and/or molecular sensors toward the development of cancer cell selective agents. This proof-of-concept study now paves the way for utilization of β-gal activity in glioblastoma cells along with NIR and two-photon absorbing PDT agents for the detection and treatment of deep tumors in animal models, and clinical studies in the future. Our work along these lines will be disclosed in due course.

## Experimental Section

### Synthesis

#### Synthesis of **1**

Commercially available resorufin (350 mg, 1.64 mmol) was dissolved in dry DMF (14 mL) at room temperature. Acetobromo-α-bromo galactose (1.35 g, 3.28 mmol) was added portion-wise, and the resulting mixture was stirred overnight at 60 °C. The reaction mixture was then diluted with ethyl acetate (200 mL) and washed with brine. Organic layers were combined and dried with the addition of Na_2_SO_4_. The crude solid was obtained with the evaporation of solvent under reduced pressure and then purified by silica gel column chromatography (Hex/EtOAc 1:2) to give the title compound as orange crystals (570 mg, 64%). ^1^H NMR (500 MHz, CDCl_3_): δ 7.73 (d, *J* = 8.8 Hz, 1H), 7.42 (d, *J* = 9.8 Hz, 1H), 7.03–6.96 (m, 2H), 6.84 (dd, *J* = 9.8, 2.0 Hz, 1H), 6.30 (d, *J* = 2.1 Hz, 1H), 5.56–5.48 (m, 2H), 5.20–5.13 (m, 2H), 4.25 (dd, *J* = 10.6, 6.7 Hz, 1H), 4.21–4.14 (m, 2H), 2.20 (s, 3H), 2.10 (s, 3H), 2.09 (s, 3H), 2.03 (s, 3H). ^13^C NMR (126 MHz, CDCl_3_): δ 186.23, 170.29, 170.10, 170.02, 169.25, 159.84, 149.51, 146.90, 145.10, 134.78, 134.69, 131.60, 129.56, 114.97, 106.95, 103.43, 98.89, 71.58, 70.62, 68.29, 66.74, 61.45, 20.70, 20.61, 20.59, 20.54. HRMS *m*/*z* calcd. for C_26_H_26_NO_12_: 544.1455. Found: 544.1458 [M + H]^+^.

#### Synthesis of **2**

**1** (250 mg, 0.46 mmol) was dissolved in EtOH (40 mL). Iodine (300 mg, 1.18 mmol) was added to the reaction medium. Further, iodic acid (47.5 mg, 0.27 mmol) solution in 2 mL of water was added dropwise, and the reaction mixture was refluxed for 4 h (red spot that belongs to the product should be closely monitored). Upon monitoring the reaction progress by TLC, the mixture was cooled to RT, and the solvent was evaporated under a vacuum. Residual solids were disolved with EtOAc (130 mL) and washed with saturated sodium thiosulfate solution. The organic layer was separated and dried with Na_2_SO_4_, and the solvent was evaporated under reduced pressure. The crude product was purified by silica gel column chromatography (Hex/EtOAc 1:1) to give the title compound as red crystals (163 mg, 53%). ^1^H NMR (500 MHz, CDCl_3_): δ 7.79 (d, *J* = 8.8 Hz, 1H), 7.44 (d, *J* = 9.7 Hz, 1H), 7.12–7.03 (m, 3H), 5.56 (dd, *J* = 10.4, 7.9 Hz, 1H), 5.52 (d, *J* = 3.5 Hz, 1H), 5.24 (d, *J* = 7.9 Hz, 1H), 5.17 (dd, *J* = 10.4, 3.5 Hz, 1H), 4.27–4.19 (m, 3H), 2.22 (s, 3H), 2.14 (s, 3H), 2.10 (s, 3H), 2.04 (s, 3H). ^13^C NMR (126 MHz, CDCl_3_): δ 180.76, 170.52, 170.25, 170.15, 169.40, 160.42, 151.02, 145.61, 145.59, 134.90, 132.72, 131.69, 129.87, 115.81, 103.65, 98.90, 85.94, 71.88, 70.73, 68.43, 66.96, 61.79, 21.13, 20.87, 20.79, 20.69. HRMS *m*/*z* calcd. for C_26_H_25_NO_12_I: 670.0421. Found: 670.0422 [M + H]^+^.

#### Synthesis of **RB-1**

**2** (100 mg, 0.15 mmol) was dissolved in methanol (6 mL), and it was cooled to 0 °C. NaOMe solution (0.16 mL, 4.4 M) was added dropwise, and the reaction mixture was stirred for 1 h. Upon completion of the reaction, the mixture was neutralized with Amberlite IR-120 plus until pH = 7 was reached. Amberlite was filtered off, and the filtrate was evaporated. The resulting crude product was purified by RP-HPLC (gradient of acetonitrile 1–90% in water), and the solvents were evaporated to give the title compound as red crystals (63.9 mg, 85%). ^1^H NMR (400 MHz, DMSO-*d*_6_): δ 7.85 (d, *J* = 8.9 Hz, 1H), 7.57 (d, *J* = 9.7 Hz, 1H), 7.22 (d, *J* = 2.6 Hz, 1H), 7.16 (dd, *J* = 8.9, 2.5 Hz, 1H), 6.99 (d, *J* = 9.7 Hz, 1H), 5.30 (d, *J* = 5.1 Hz, 1H), 5.17 (d, *J* = 7.6 Hz, 1H), 4.95 (d, *J* = 5.7 Hz, 1H), 4.73 (t, *J* = 5.5 Hz, 1H), 4.60 (d, *J* = 4.6 Hz, 1H), 3.76 (t, *J* = 6.2 Hz, 1H), 3.74–3.71 (m, 1H), 3.67–3.62 (m, 1H), 3.58 (dd, *J* = 11.0, 5.6 Hz, 1H), 3.52 (dd, *J* = 11.1, 5.1 Hz, 1H), 3.47 (dd, *J* = 8.6, 5.4 Hz, 1H). ^13^C NMR (101 MHz, DMSO-*d*_6_): δ 166.13, 147.17, 136.82, 131.01, 130.18, 120.69, 117.32, 116.80, 114.41, 101.35, 88.16, 86.35, 70.63, 61.33, 58.70, 55.77, 53.70, 45.90. HRMS *m*/*z* calcd. for C_18_H_17_NO_8_I: 501.9999. Found: 501.9999 [M + H]^+^.

### HPLC Purification and Analyses

**RB-1** was purified with Dionex Ultimate 3000 by using a *semipreperative* C18 HPLC column. (Thermo Scientific Hypersil Gold, 250 × 10 mm, 5 μm) The eluents were Milli Q water (with 0.1% TFA) and acetonitrile (with 0.08% TFA). The purified product was analyzed by using a Thermo Scientific Hypersil Gold C18 analytical column (4.6 × 150 mm, 3 μm). A gradient method was used starting from 1% acetonitrile to 90% acetonitrile in 30 min.

To monitor the reaction of **RB-1** with β-gal, HPLC analysis was performed using an RP-HPLC System with UV–vis detection and a reversed-phase C18 column (4 μm, 4.6 × 150 mm). The data collection and analysis were carried out using the Chemstation software. The oven temperature was kept at 25 °C. The injection volume was adjusted to 20 μL, and the flow rate was 1.0 mL/min. The detection wavelength was 480 nm. The separation program of gradient elution was water with 0.1% TFA and acetonitrile.

#### Fluorescence Quantum Yield

The fluorescence quantum yields (Φ_F_) for the samples were calculated by using the formula given below:
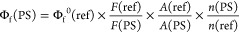
*F* represents the integrated area under the fluorescence curve, *n* is the refractive index of the solvent used, and *A* is the absrobance value collected at 570 nm for each sample, which represents the absorption maximum of the reference compound. PS defines either **RB-1** or **RB1** + β-gal. As a reference compound, resorufin sodium salt was used (Φ_F_ = 0.74).^49^

### Chemical Detection of Singlet Oxygen

Singlet oxygen quantum yield of **RB-1** was calculated by following our previously reported article.^24^ A 595 nm LED (9.83 mW/cm^2^) was used as a light source.

### Cell Culture Studies

#### Cell Culture and Treatments

Human glioblastoma (U-87 MG) and healthy mouse fibroblast (NIH/3T3) cells were cultured in DMEM high glucose supplemented with 10% fetal bovine serum (FBS), 1% penicillin/stremptomycin, 0.5% amphotericin B, and 2 mM glutamine at 37 °C with 5% CO_2_. For PDT application, cells were treated with varying concentrations of **RB-1** (0.5–32 μM) for 0.5–4 h followed by 595 nm LED (9.83 mW/cm^2^) illumination for 2 h without washing the agent, then 24 h of rest in the dark. For the dark toxicity, cells were subjected to the same treatment under identical conditions without LED illumination and incubated for 24 h without washing prior to cell viability analysis.

#### Cell Viability

Cells treated with increasing concentrations of **RB-1** then conventional MTT (3-[4,5-dimethylthiazol-2-yl]-2,5-diphenyl-tetrazolium bromide) analysis were held as described, previously.^15^ Absorbance values were normalized as the percentages of DMSO-treated controls. IC_50_ values were obtained by using nonlinear regression analysis (GraphPad Prism 8.02, GraphPad Software Inc.; *n* = 6).

#### Confocal Imaging

Cells (1 × 10^4^) were seeded on glass-bottom confocal dishes (35 mm) and then treated with **RB-1** (2 μM, 0.5 and 4 h) at 37 °C. Cells were washed three times with 1× PBS and then fixed with 4% paraformaldehyde for 15–20 min at RT. After washing steps, cells were treated with Hoechst 33342 (1 μg/mL) and LysoTracker Yellow HCK-123 (75 nM in PBS), and confocal images were taken at defined excitation/emission wavelengths of 361/497 nm (Hoechst), 465/535 (Lysotracker), and 580/600 (**RB-1**) by Zeiss LSM 900 CLSM, respectively (40×; *n* = 3)

#### Intracellular ROS Generation

Cells were incubated with the IC_50_ dose of **RB-1** (4 h) in the dark, then exposed to 595 nm LED light for 2 h with and without scavengers for ROS (N-acetylcysteine, NAC), singlet oxygen (sodium azide, NaN_3_), superoxide anion (Tiron), or hydroxyl radical (mannitol), then incubated up to 24 h in the dark before running an MTT analysis. For confocal imaging, cells were incubated with **RB-1** (4 h), then exposed to LED light with and without NAC or NaN_3_ for 2 h. Cells were washed twice with 1× PBS, then treated with DCFH-DA (20 μM), PI (10 μg/mL), and Hoechst 33342 (2 μg/mL) in serum free media for 30–45 min. After washing steps, confocal images were obtained at 488/535 nm (ex/em), 550/617 nm (ex/em), and 361/497 nm (ex/em) wavelengths for DCF, PI, and Hoechst (10×; *n* = 6).

#### Cell Death Mechanism

Cells were incubated with **RB-1** by following the PDT protocol as previously described, then kept at 37 °C for 0.5 h. Cells were treated with AO (1 μg/mL) and EtBr (1 μg/mL) for 30 min at 37 °C, after washing the cells (PBS, two times). Images were obtained at 500/525 (ex/em) and 530/617 nm (ex/em) wavelengths with a confocal microscope (10×; *n* = 6)

## Supporting Information Available

Synthetic protocols, NMR and HR-MS spectra, chemical detection ^1^O_2_, experimental details of photophysical characterization with additional measurements, experimental details of *in vitro* experiments, and additional cell culture experiments (PDF)

## Abbreviations

PDT: photodynamic therapy
PS: photosensitizer
aPS: activatable photosensitizer
ROS: reactive oxygen species
ICT: intramolecular charge transfer process
β-gal: β-galactosidase
GBM: glioblastoma
ADMDA: 2,2′-(anthracene-9,10-diyl)bis(methylene)dimalonic acid
PBS: phosphate buffer saline
ISC: intersystem crossing
EtOH: ethanol
DMSO: dimethyl sulfoxide
MTT: 3-(4,5-dimethylthiazolyl-2)-2,5-diphenyltetrazolium bromide
PI: propidium iodide
DCFH_2_-DA: 2′,7′-dichloro-fluorescein diacetate
NAC: N-acetyl cysteine
NaN_3_: sodium azide
LED: light emitting diode
AO: acridine orange
EtBr: ethidium bromide
IC_50_: half maximal inhibitory concentration
