# Supplementary material for: Locked and Loaded: β-Galactosidase Activated Photodynamic Therapy Agent Enables Selective Imaging and Targeted Treatment of Glioblastoma Multiforme Cancer Cells
Source: ACS Appl Bio Mater. 2022 Aug 31;5(9):4284–93. doi: 10.1021/acsabm.2c00484 (PMC9490748; doi:10.1021/acsabm.2c00484)
Supplement: Supplementary file 1 — mt2c00484_si_001.pdf [file mt2c00484_si_001.pdf]

Supporting Information

Locked and loaded: β-galactosidase activated photodynamic therapy agent enables selective imaging and targeted treatment of glioblastoma multiforme cancer cells

Toghrul Almammadov,[a‡] Zubeyir Elmazoglu,[b‡] Gizem Atakan,[b] Dilay Kepil [b], Guzide Aykent,[b] Safacan Kolemen[a,c,d]* and Gorkem Gunbas[b]*

[a]Department of Chemistry, Koç University, Rumelifeneri Yolu, 34450 Istanbul, Turkey
[b]Department of Chemistry, Middle East Technical University (METU), 06800 Ankara, Turkey
[c]Surface Science and Technology Center (KUYTAM), Koç University, 34450 Istanbul, Turkey
[d]Boron and Advanced Materials Application and Research Center, Koç University, 34450 Istanbul, Turkey

‡ These authors contributed equally.

E-mail: skolemen@ku.edu.tr, ggunbas@metu.edu.tr

1. General

Reagents used in the experiments were commercially available and purchased from Sigma Aldrich. Mbraun MBSPS5 solvent drying system was used to prepare the dry solvents. Bruker Avance III Ultrashield (500 MHz) spectrometer was used for getting ^{1}H and ^{13}C-NMR spectra. For preparing the NMR samples deuterated solvents $CDCl_3$ or DMSO-d_6 were used, which both contain TMS (trimethylsilane) as an internal reference. Chemical shifts were reported in terms of parts per million (ppm), which are arranged according to the internal reference (TMS). Silica Gel 60 (Merck 230-400 mesh) and thick-wall columns were used for purifying the compounds. Reaction progress was monitored by thin layer chromatography (TLC - Merck Silica Gel 60 F254) under a UV lamp. Shimadzu UV-3600 UV-VIS-NIR spectrophotometer was used for acquiring the electronic absorption spectra of the samples. Agilent Cary Eclipse fluorescence spectrophotometer was used for recording the emission spectra. Waters Synapt G1 High-Definition spectrometer was used to collect the mass data of the samples.

2. HPLC Analyses

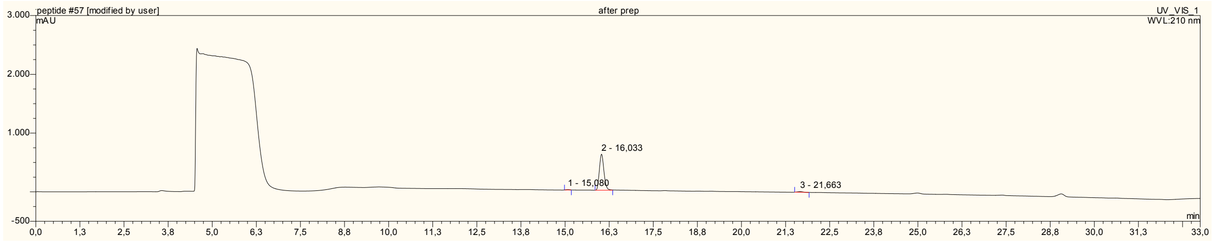

No.	Ret.Time	Peak Name	Height	Area	Rel.Area
	min		mAU	mAU*min	%
1	15.08	n.a.	6.806	0.721	0.76
2	16.03	**RB-1**	612.493	91.985	**97.22**
3	21.66	n.a.	10.556	1.911	2.02
Total:			629.855	94.617	100.00

Figure S1. HPLC chromatogram and relative areas of peaks at defined retention times. (The peak at retention time 5 min is dead time and acetic acid which was the solvent used for sample preparation). Detection wavelength = 210 nm.

For proving the enzymatic reaction between **RB-1** and β-gal, each of analyte, including Res-I were injected to HPLC with UV-Vis detection and a reversed-phase C18 column (4 µm, 4.6 × 150 mm) using a program of acetonitrile (Phase A) and water with 0.1 % TFA (Phase B), which is given below:

Time/min	Phase A/%	Phase B/%
0	10.0	5.0
7.0	5.0	95.0
13.0	5.0	95.0
16.0	95.0	5.0

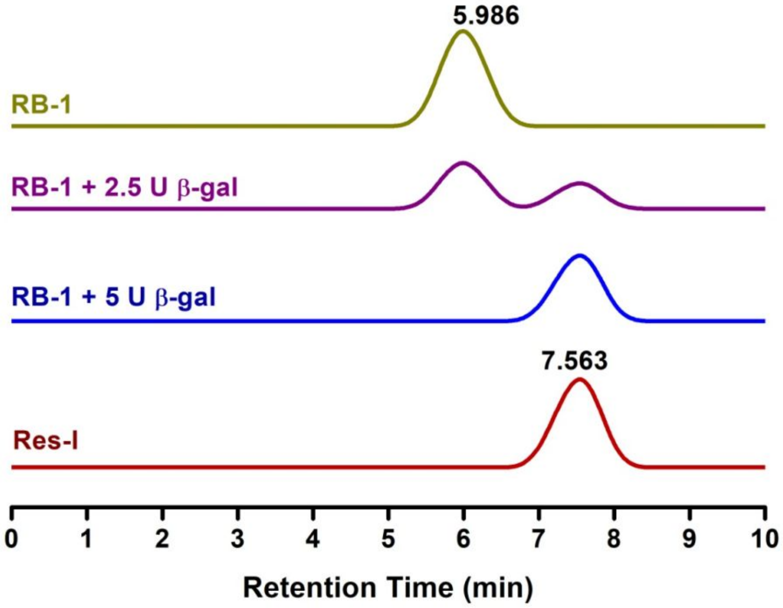

Figure S2. HPLC chromatograms of **RB-1** before and after treatment with either 2.5 U or 5 U β-gal at 37 °C and Res-I. (Detection wavelength = 480 nm).

3. Photophysical Characterization

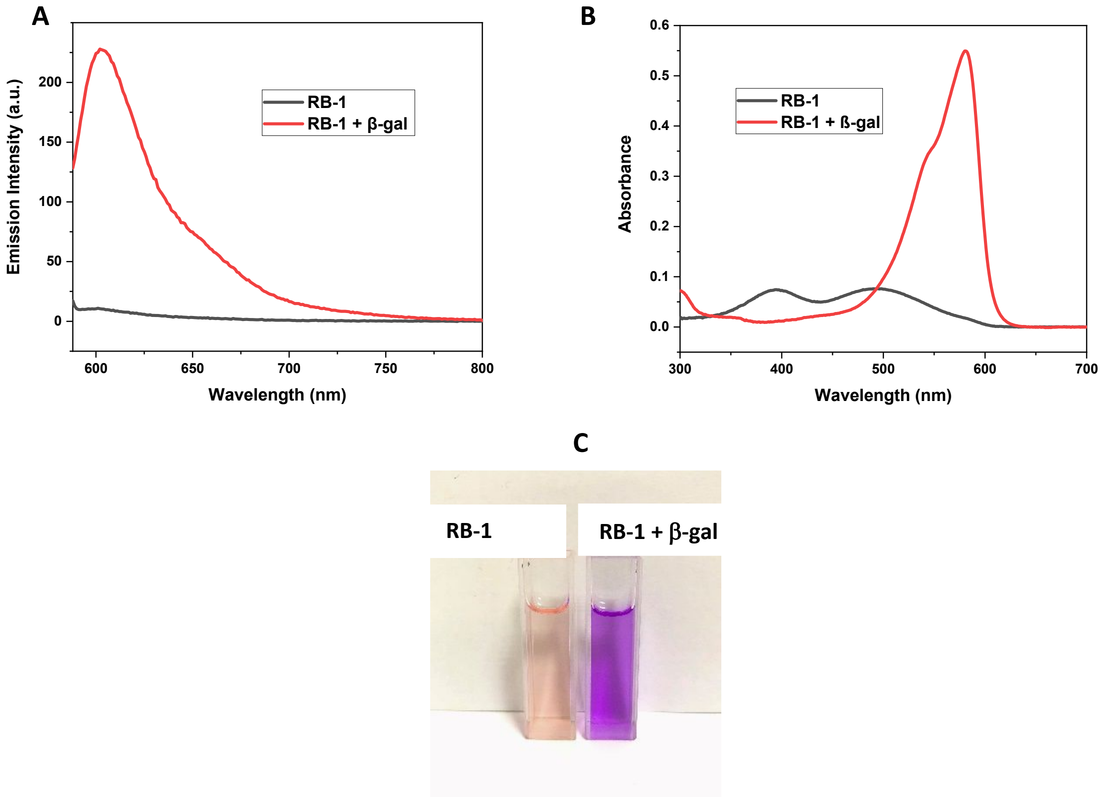

Figure S3. Absorption (a) and emission (b) spectra of **RB-1** before and after β-gal (5U) treatment at 37°C in PBS (1% DMSO, pH 7.4), and (c) corresponding color change upon addition of the enzyme.

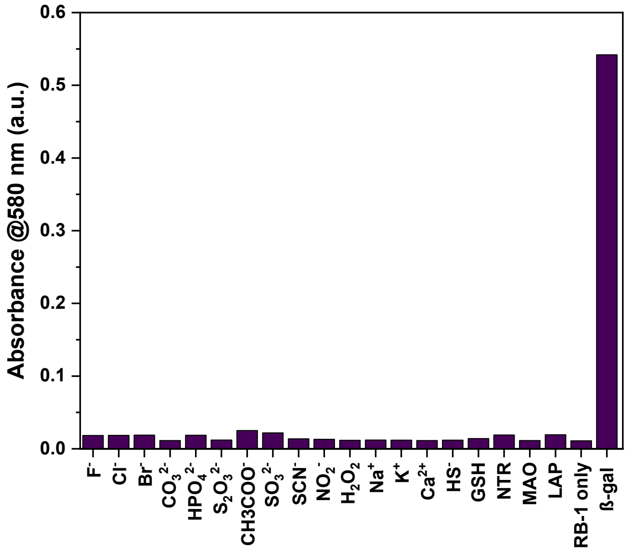

Figure S4. Absorption signal of **RB-1** (10 µM) itself and after addition of different analytes (100 µM each) at 580 nm.

4. Cell culture

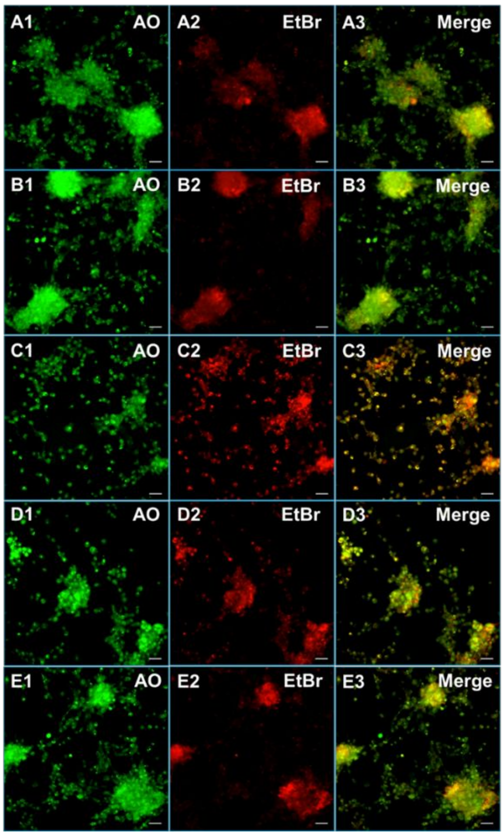

Figure S5. Dual AO/EtBr staining of U-87MG cells treated with DMSO (0.2%) **(A1-3)** or **RB-1** (7.7 µM) at dark for 4 h **(B1-3)**, illuminated with LED light (595 nm) for 2 h in the absence **(C1-3)** or presence of NAC (10 mM) **(D1-3)** or NaN_3 (10 mM) **(E1-3)** for 2 h. Green: AO, Red: EtBr. AO: acridine orange, EtBr: ethidium bromide. Scale bar: 50 µm.

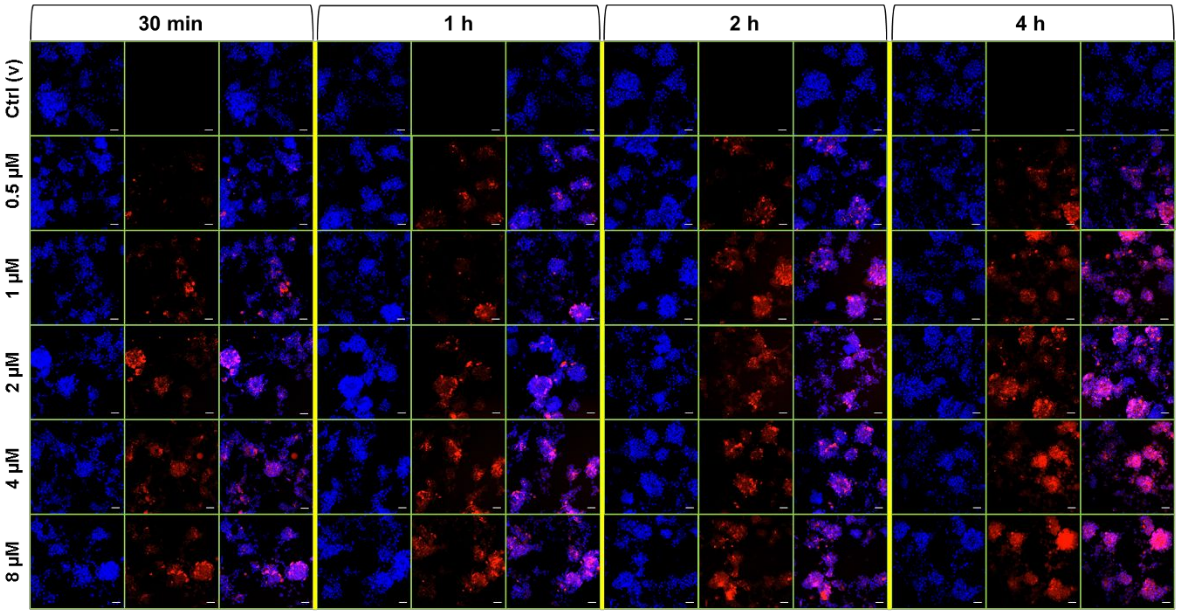

Figure S6. Confocal images of varying concentrations of **RB-1** (0.5 μM – 8 μM) in U-87MG cells after 0.5, 1, 2 or 4 h. Blue: Hoechst 33342; Red: **RB-1**. Scale bar: 50 μm.

5. NMR Spectra

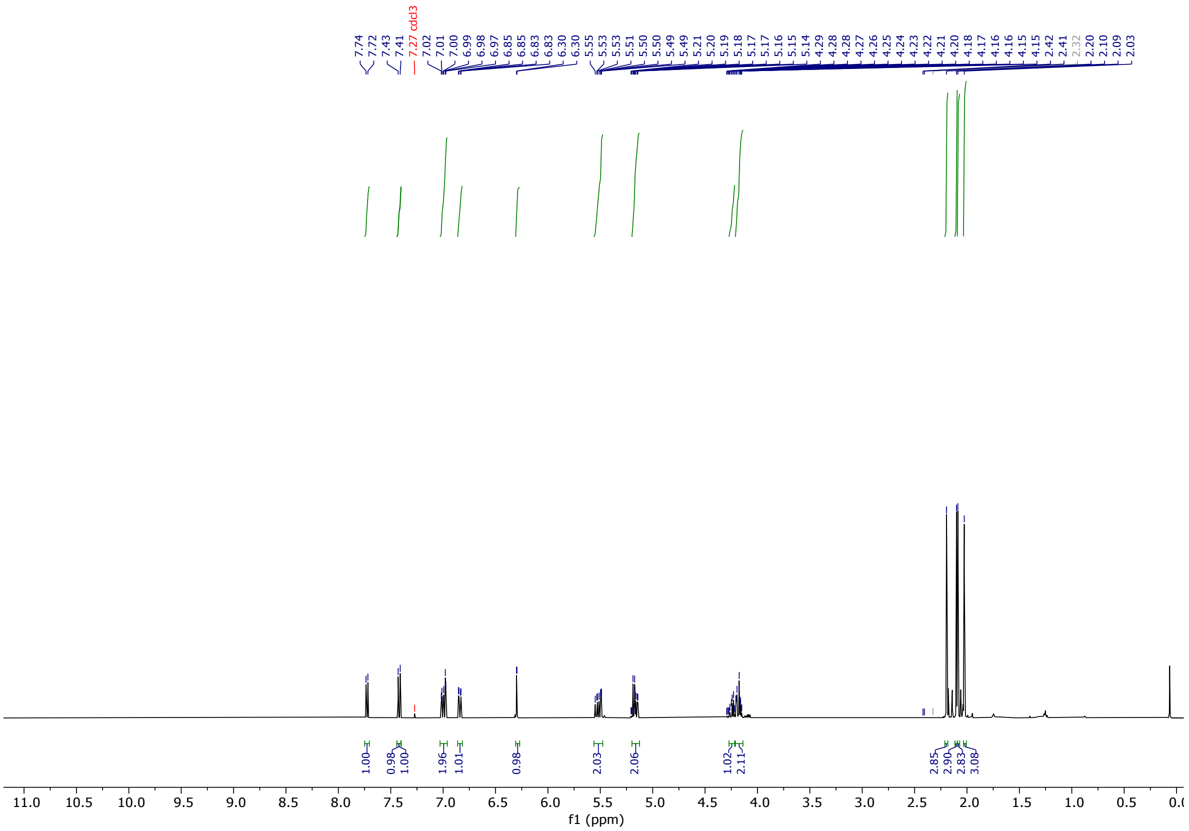

Figure S7. ^{1}H NMR spectrum of compound **1.**

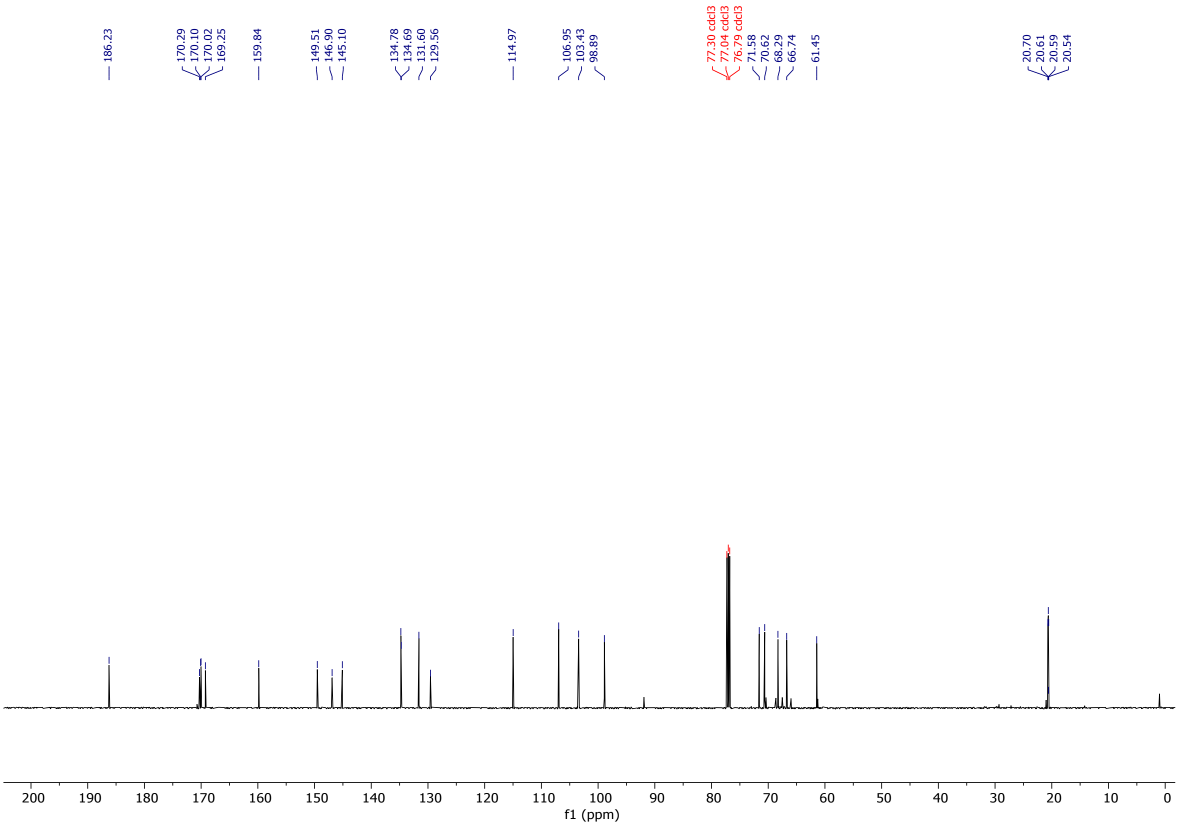

Figure S8. ^{13}C NMR spectrum of compound **1.**

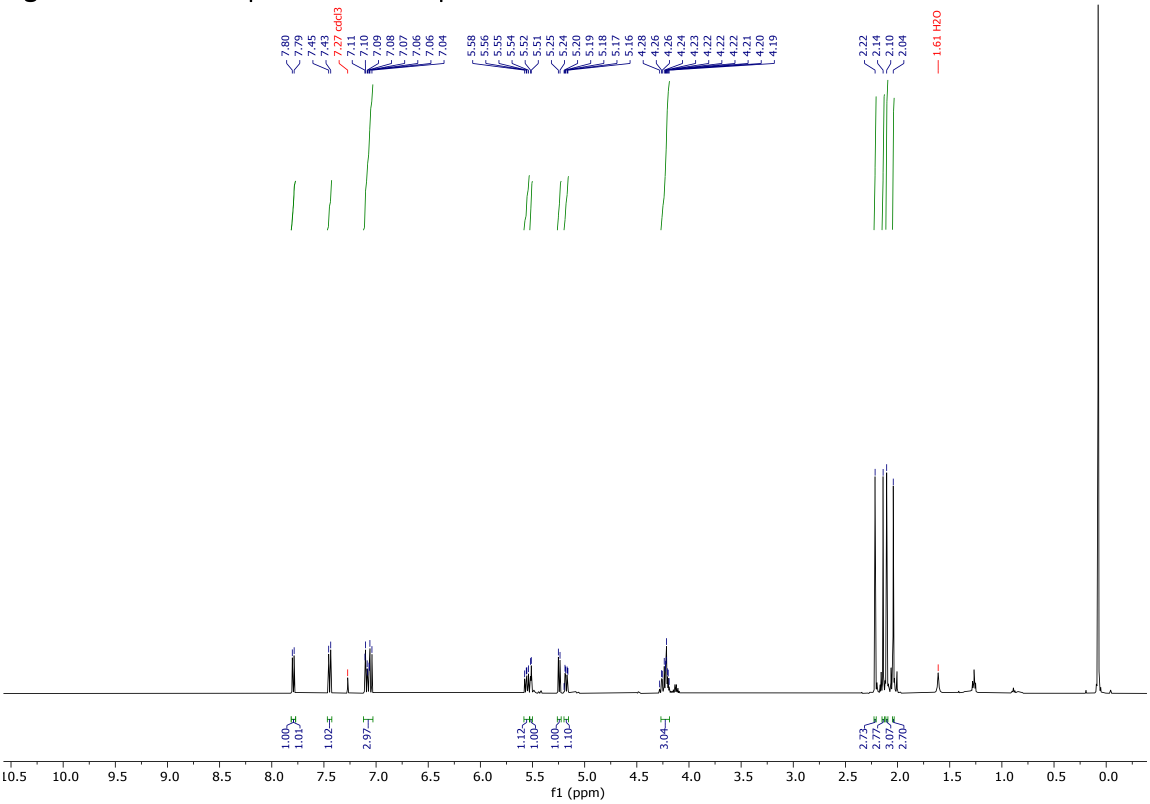

Figure S9. ^{1}H NMR spectrum of compound **2.**

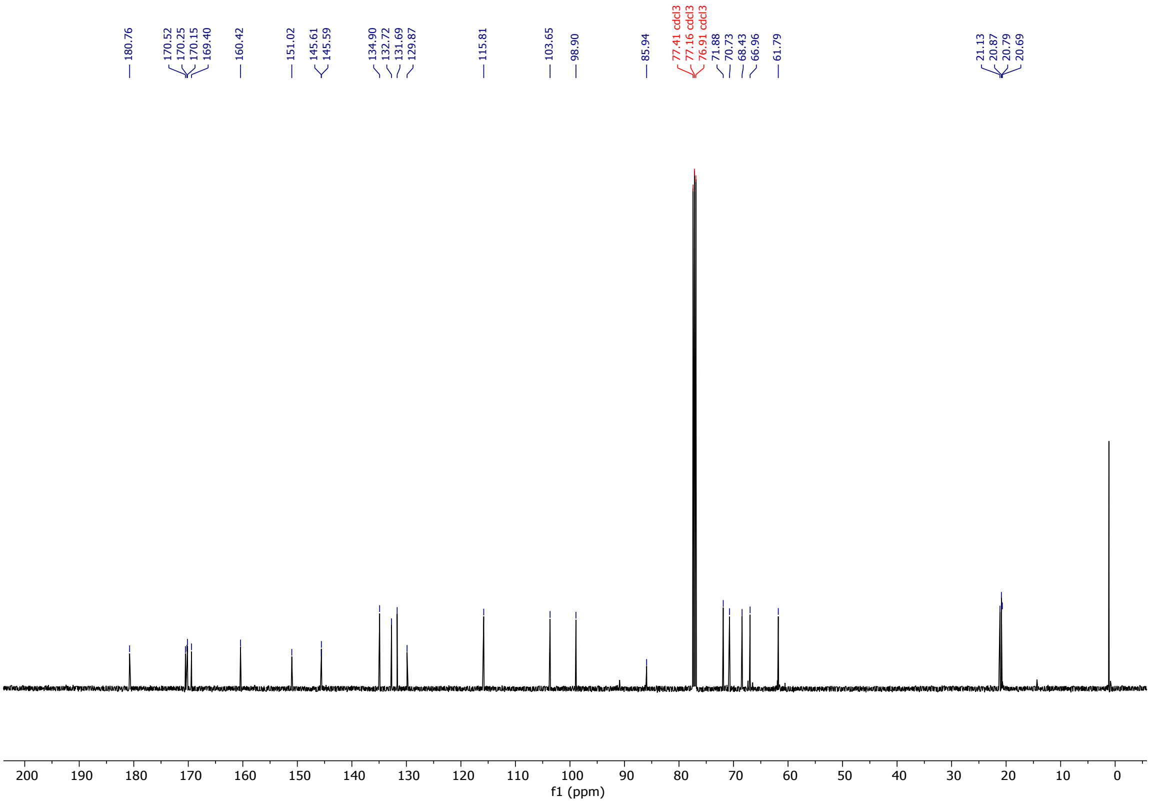

Figure S10. ^{13}C NMR spectrum of compound **2.**

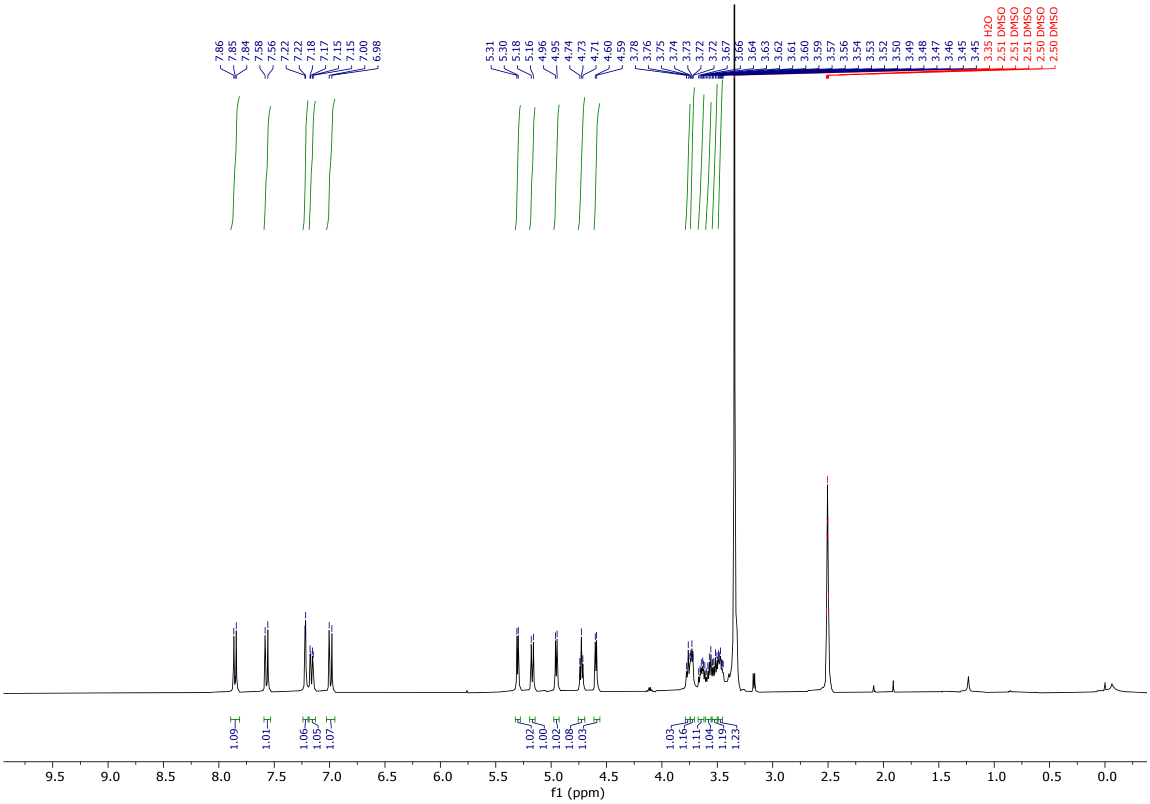

Figure S11. ^{1}H NMR spectrum of compound **RB-1.**

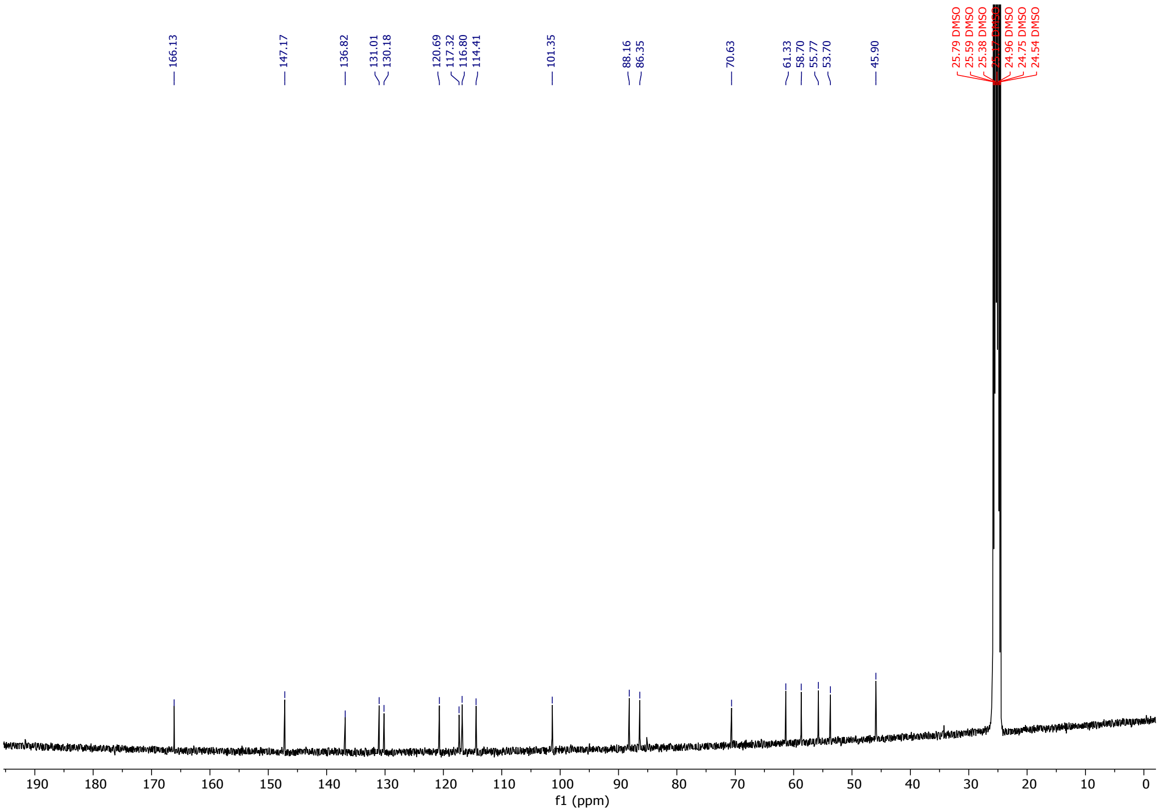

Figure S12. ^{13}C NMR spectrum of compound **RB-1.**

6. HRMS Spectra

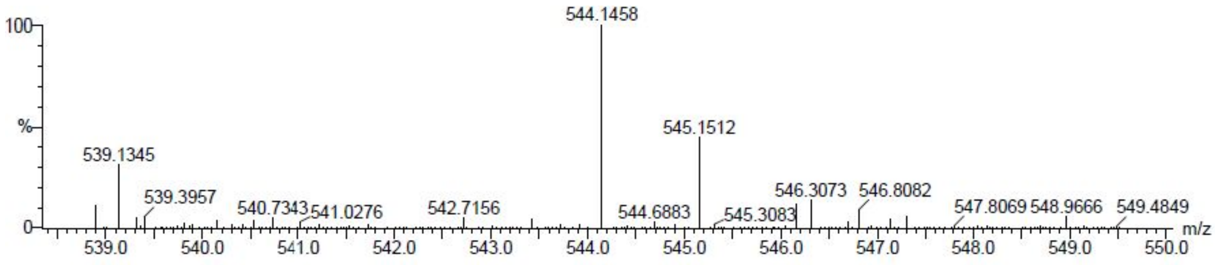

FigureS13. HRMS spectrum of compound **1**.

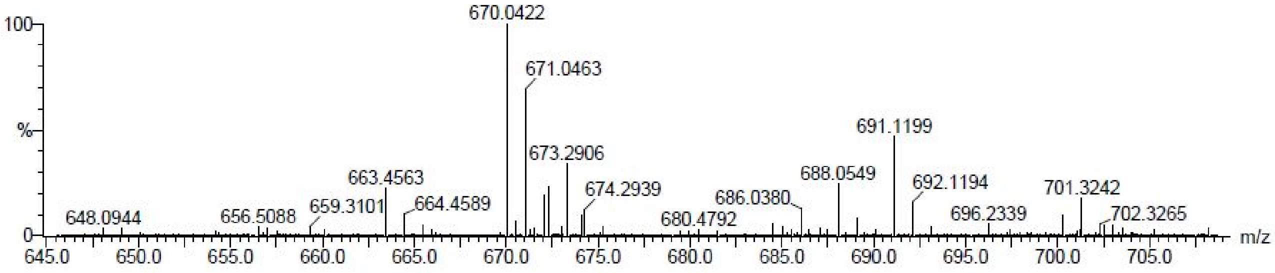

FigureS14. HRMS spectrum of compound **2**.

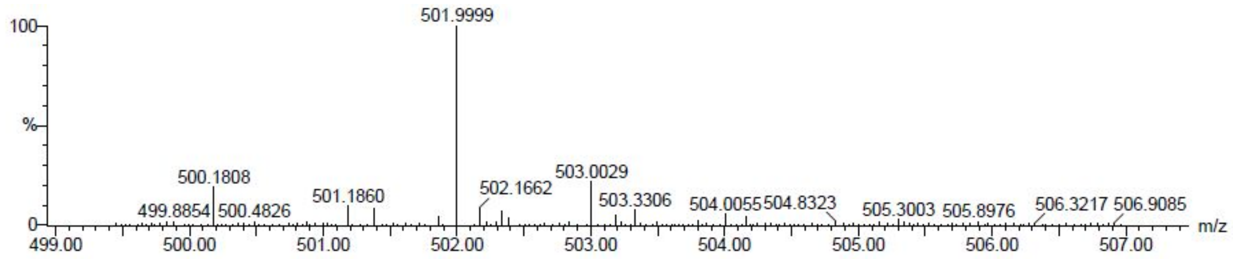

Figure S15. HRMS spectrum of compound **RB-1**
